# Co-Amoxiclav as empiric treatment of UTI in children: importance of surveillance in ensuring optimal empiric treatment choice

**DOI:** 10.1007/s11096-021-01318-y

**Published:** 2021-08-22

**Authors:** James Trayer, Michael Horgan, Anna-Rose Prior, Martin Ryan, Montasser Nadeem

**Affiliations:** 1 Department of Paediatric, Children’s Health Ireland at Tallaght University Hospital, Dublin, Ireland; 2 Department of Microbiology, Tallaght University Hospital, Dublin, Ireland; 3 Department of Radiology, Tallaght University Hospital, Dublin, Ireland; 4 Trinity College Dublin, Dublin, Ireland

**Keywords:** Antibiotic resistance, General Paediatric, Nephrology, Urinary tract infection

## Abstract

*Background* Urinary tract infections are common and require prompt treatment. *Objective* To examine the resistance rates of co-amoxiclav in children with urinary tract infection and whether antimicrobial resistance is influenced by other variables. *Methods* The records and antibiotic susceptibility data of 209 patients admitted with symptomatic urinary tract infection between January 2018 and December 2019 were reviewed. *Results* We examined 209 patients [mean (SD) age 23.73 (32.86) months], of whom 176 (84.2%) had first urinary tract infection. *Escherichia coli* was isolated in 190 (90.1%). Uropathogens were sensitive to co-amoxiclav in 47.8% of patients and gentamicin in 95.2%. Combined co-amoxiclav with gentamicin demonstrated antimicrobial sensitivity in 96.2%. Antimicrobial resistance was associated with longer hospital stay (*p*-value < 0.02). An association was identified between co-amoxiclav resistance and recurrent urinary tract infections. Uropathogens were resistant to co-amoxiclav in 80/176 (45.5%) and 29/33 (87.9%) patients with first and recurrent urinary tract infections, respectively (*p*-value 0.001). No link was observed between antimicrobial resistance and atypical urinary tract infection. *Conclusion* Approximately half of children in this cohort had urinary tract infection due to uropathogens resistant to co-amoxiclav. Co-amoxiclav resistance is associate with recurrent infections and longer hospital stays. A combination of co-amoxiclav and gentamicin demonstrates > 96% susceptibility.

## Impacts on practice

- More than 50% of children presenting with urinary tract infection have uropathogens resistant to co-amoxiclav.
- Resistance to co-amoxiclav is associated with recurrent urinary tract infection and longer hospital stay in children and adolescents with urinary tract infection.
- Sensitivity to co-amoxiclav improves from 47.8% to > 96% when used in combination with gentamicin.

## Introduction

Urinary Tract Infections (UTI) occur in up to 6–8% of febrile infants and young children, with *Escherichia coli* (*E*. *coli*) being the commonest uropathogen [1]. Accurate diagnosis of UTI in children is important as there is a risk of renal scarring if left untreated. Conversely, the treatment of asymptomatic bacteriuria exposes patients to the potential adverse effects of antimicrobials and accelerates the development of resistance.

Urine culture is the gold standard for identifying the causative uropathogen and performing antimicrobial sensitivities to guide treatment. These results may take up to 48 h so the choice of empiric antibiotic therapy during this time is important. Numerous guidelines exist regarding this [2–6]. NICE guidelines recommend that for children > 3 months old co-amoxiclav should only be used in combination with other drugs or if known to be susceptible [2]. American Academy of Paediatrics (AAP) guidelines recommend antimicrobial prescribing based on local antibiotic susceptibility data, however they make no recommendation regarding the empiric use of antibiotics in combination [3].

Unnecessarily antibiotic exposure could lead to emergence of antibiotic resistance. Indeed this occurs with commonly used empiric antibiotics, with a hypothesis that antibiotic resistance can occur in children using empiric antibiotics for recurrent UTI.

### Aim of the study

The aim of this study was to examine the resistance rates of uropathogens to empiric co-amoxiclav as monotherapy and in combination with gentamicin. We also aimed to investigate whether there is a link between co-amoxiclav resistance and other variables such as recurrent UTI, patients’ age at presentation, gender, and the presence of atypical UTI or radiological abnormalities in children and adolescents with UTI abnormalities.

### Study approval

This was a retrospective review of the electronic patients records of patients admitted with UTI. Approval was obtained to perform this review.

## Methods

A cohort of 209 patients with symptomatic UTI admitted to a paediatric hospital between January 2018 and December 2019 was identified from electronic patient records. Children with renal hypodysplasia, renal agenesis, multicystic dysplastic kidney, polycystic kidney disease, ureteropelvic junction obstruction or neural tube defects were excluded. A UTI was defined as the presence of typical symptoms in a child with a pure growth of an organism with > 10^5^ colony forming units per millilitre on urine culture. Antibiotic susceptibility data were obtained from the laboratory information system. Radiologic imaging results were also reviewed in order to identify any potential association between urinary tract abnormalities and antimicrobial resistance rates. Abnormal dimercaptosuccinic acid scintigraphy (DMSA) was defined as the presence of renal scarring or significant imbalance in renal uptake. Micturating cystourethrogram (MCUG) was defined as abnormal if vesicoureteral reflux (VUR) or renal tract obstruction was identified. Atypical and recurrent UTI were defined as per NICE guidelines (2).

Using SPSS version 27, summary measures were calculated and are reported as mean and standard deviation (SD). One-way ANOVA was used to explore the differences between means. Univariate and multivariate logistic regression models were used to estimate the association between the variables and the outcome. A *p*-value < 0.05 was considered statistically significant.

## Results

We examined 209 patients with UTI, the demographic data is presented in Table 1. The mean (SD) age was 23.73 (32.86) months, [range 5 days–13.93 years] and length of stay was 3.42 (2.13) days. Of 209 patients, 120 (57.4%) were female, 190 (90.1%) had *E*. *coli* identified as the causative pathogen and 176/209 (84.2%), experienced their first UTI. In this cohort of patients, over half (109 of 209 (52.2%)) had UTI due to uropathogens resistant to co-amoxiclav. In this cohort of patients, a review of sensitivities to alternative antibiotics demonstrated gentamicin sensitivity in 199/209 (95.2%), cefuroxime sensitivity in 194/209 (92.8%), cefalexin sensitivity in 189/209 (90.4%), nitrofurantoin sensitivity in 207/209 (99.0%) and trimethoprim sensitivity in 142/209 (67.9%). Notably, when co-amoxiclav in combination with gentamicin was analysed, overall susceptibility to both agents was 96.2% (201/209). Similarly, sensitivity to cefuroxime and cephalexin in combination with gentamicin was demonstrated in 207/209 (99%) and 206/208 (99%), respectively.

**Table 1:** Patient demographics including type of UTI and radiological findings

	Number	Percent (%)
Number of patients	209	
Female	120	57.4
*E.* *coli* identified as the causative pathogen	190	90.1
First UTI	176	84.2
Recurrent UTI^a^	33	15.8
Atypical UTI^a^	29	13.9
UTI due to uropathogens resistant to co-amoxiclav	109	52.2
Renal ultrasound scan		
Normal	165	78.9
Abnormal	25	11.9
Pyelonephritis	19	9.1

^a^Atypical and recurrent UTI were defined as per NICE guidelines[2]

Microbial resistance to co-amoxiclav was associated with increased length of hospital stay with a mean (SD) of 3.9 (2.9) days compared to 3.1 (1.8) days in those who were susceptible (*p*-value < 0.02). There was no association between co-amoxiclav resistance and patients’ age (*p*-value 0.52). Moreover, uropathogens were sensitive to co-amoxiclav in 43 of 89 (48.3%) male patients and 57 of 120 (47.5%) female individuals, with no association being reported between co-amoxiclav resistance and patients’ gender (p value 0.91).

In this cohort of patients, a link between resistance to co-amoxiclav and recurrent UTI has been reported. While the majority [29 of 33 (87.9%)] of individuals with recurrent UTI had uropathogens resistant to co-amoxiclav, approximately less than half [80 of 176 (45.5%)] of patients with first UTI experienced the same resistance (*p* value 0.001). However whether UTI is typical or atypical has no impact on the antimicrobial resistance (*p* value 0.11). Uropathogens were resistant to co-amoxiclav in 98 of 180 (54.4%) patients with uncomplicated UTI caused by *E*. *coli*. However this resistance was documented in 11 of 29 (37.93%) patients with atypical UTI caused by *E*. *coli* and non-*E*. *coli* uropathogens in 10 and 19 patients, respectively.

In this cohort, no statistically significant difference in co-amoxiclav sensitivities was observed between children with normal radiological imaging and those with renal tract abnormalities detected on ultrasound scans. Of 209 patients who underwent renal ultrasound scan (RUSS), uropathogen resistance to co-amoxiclav was reported in 87/165 (52.3%) patients with normal RUSS, 12/25 (48%) with abnormal RUSS and 10/19 (52.6%) with pyelonephritis (*p* value 0.37).

## Discussion

Increasing antimicrobial resistance rates in children with UTI has been reported in the literature [7–11]. In previous studies in paediatrics with UTI, Stuliz et al. demonstrated an increasing trend in uropathogen resistance to commonly used empiric antibiotics such as amoxicillin or amoxicillin/clavulanic acid [8]. Previous studies have demonstrated significant global variation in resistance rates [8, 9] though the overall prevalence of resistance is increasing [9]. In children admitted with urinary tract infection during a 12-year study period, high resistance rates have been noted for common empiric antibiotics, such as amoxicillin and cotrimoxazole [10]. Nevertheless, the association between unnecessarily antibiotic exposure and the emergence of antibiotic resistance has been reported [11]. It has been reported that up to 80% of antibiotics used in Europe are prescribed at primary care level [11].

In this cohort of patients, 52.2% had UTI due to uropathogens resistant to co-amoxiclav. However, when co-amoxiclav used in combination with gentamicin, the overall susceptibility to both agents increased to 96.2%. Whether co-amoxiclav is an appropriate monotherapy antibiotic in children with UTI is explored in the available guideline [2]. In accordance with NICE guideline [2], in this group of patients with UTI, we observed that co-amoxiclav should be administered as monotherapy only in the context of known sensitivities, otherwise it should be given in combination where treatment is empiric. Moreover, our finding also supports that local antibiotics should be considered in prescribing antibiotics, as it was previously recommended [3].

The results from this cohort of patients support our hypothesis that there is a link between recurrent UTI and the microbial resistance to co-amoxiclav. Moreover, the association between increased length of stay and resistance to co-amoxiclav suggests that the initial empiric choice of antibiotic may have been inappropriate.

## Conclusion

In conclusion, in this cohort of patients, approximately one in two children with UTI have uropathogens resistant to co-amoxiclav. However, one in 26 patients with UTI have microbial resistance to co-amoxiclav and gentamicin in combination. Notably, a link has been observed between resistance to co-amoxiclav and recurrent UTI, but not patients’ age at presentation, gender, the presence of atypical UTI or radiological abnormalities. In this group of patients, the association between increased length of stay and resistance to co-amoxiclav suggests that the initial empiric choice of antibiotic may have been inappropriate. The results from this cohort of patients highlight that the current guidelines should be considered in the management of UTI and that antimicrobial prescribing should be based on local antibiotic susceptibility data.
